# Are your injured soccer players at risk of re-injury?

**DOI:** 10.1186/1757-1146-8-S2-O34

**Published:** 2015-09-22

**Authors:** Bianca Knight, Kristy Robson, Kristen Andrews

**Affiliations:** 1 School of Community Health, Charles Sturt University, Albury, 2640 Australia

**Keywords:** Sports injury, Soccer, re-injury, rehabilitation

## Background

Injury rates in soccer are reported as one of the highest of all sports (Schmikli et al 2011). Evidence also suggests that in amateur soccer, re-injury rates are as high as 30% often resulting in more severe subsequent injuries (Ekstrand, Hägglund, and Waldén 2011). It has been suggested that premature return to play is one of the main risk factors for re-injury (Schmikli et al 2011; Junge & Dvorak 2004). Coaches are intergral to the return to play decision in amateur soccer. However, little is known on what influences coaches decisions or how these decisions are supported by interactions with health professionals, including podiatrists.

## Methods

The purpose of this study was to understand coaches' experience of return to play decisions. A qualitative design using Interpretive Phenomenological Analysis (IPA) was implemented to elicit in-depth information from five amateur soccer coaches through semi-structured interviews. IPA focuses on the subjective lived experiences of the participants and the perceptions individuals have around this experience (Finlay 2009). IPA addresses the aims of the study by illuminating the coaches' experience of return to play and highlights the issues they perceive are of importance.

## Results

Several themes were illuminated from the data, with two of particular significance to podiatrists; *coaches' subjectivity* and *limited collaboration with health professional's influences return to play decisions*. In the absence of formal return to play guidelines and variable knowledge on injury management, coaches would make subjective decisions based on value of the player or importance of the game, even if the player was not fully rehabilitated. A lack of collaboration with health professionals about injured players resulted in reliance upon the player for communicating the rehabilitation plan and progress. This influenced the coaches' decisions as the player was not always seen as a reliable source.

## Conclusions

Podiatrists may be able to impact on the high rates of re-injury in amateur soccer players if there was greater collaboration in injury rehabilitation between the health professional, player and coach. By enhancing the lines of communication this could also increase the understanding of injury rehabilitation, and reduce the subjectivity in amateur soccer coaches return to play decisions.

